# Phenazopyridine‐Induced Sulfhemoglobinemia

**DOI:** 10.1002/ccr3.73912

**Published:** 2026-11-09

**Authors:** Waldemar Irizarry, Ashton Taylor, Amaya Arora, Astha Guliani

**Affiliations:** ^1^ Northeast Ohio Medical University Rootstown Ohio USA; ^2^ Department of Internal Medicine Akron General Medical Center Akron Ohio USA; ^3^ Department of Science and Engineering Barrington High School Barrington Illinois USA

**Keywords:** co‐oximetry, dyshemoglobinemia, hypoxemia, oxidative hemolytic anemia, phenazopyridine, sulfhemoglobinemia

## Abstract

Prolonged phenazopyridine exposure can cause sulfhemoglobinemia and suspected oxidative hemolytic anemia. Persistent low pulse oximetry readings with indeterminate co‐oximetry warrant laboratory consultation. Management requires withdrawal of the offending drug and supportive care; methylene blue does not reverse sulfhemoglobinemia.

## 1 Introduction

Phenazopyridine hydrochloride is a urinary tract analgesic used for symptomatic relief of dysuria. When prescribed with antibacterial therapy for a urinary tract infection, treatment should not exceed 2 days. It is contraindicated in renal insufficiency, and usual adult doses may cause toxicity in patients with impaired renal function. Reported adverse effects include methemoglobinemia, sulfhemoglobinemia, hemolytic anemia, and renal and hepatic toxicity [1, 2].

Sulfhemoglobinemia is a rare dyshemoglobinemia that impairs functional oxygen transport. Sulfhemoglobin cannot be reduced to normal hemoglobin with methylene blue; improvement depends on removal of the offending exposure, supportive care, and replacement or turnover of affected erythrocytes [3]. Its overlap with methemoglobinemia and interference with some co‐oximeters can complicate diagnosis [3].

We describe phenazopyridine‐associated sulfhemoglobinemia with suspected oxidative hemolytic anemia in a patient with recurrent calcium oxalate nephrolithiasis. Indeterminate co‐oximetry delayed recognition of sulfhemoglobinemia, and laboratory consultation clarified the diagnosis. Clomipramine use and the patient's stone history also affected treatment decisions.

## 2 Case History and Examination

A 60‐year‐old woman presented for left ureteroscopy with laser lithotripsy, ureteral stent exchange, and kidney stone extraction. The day of presentation and admission is designated Day 0. Her history included recurrent calcium oxalate nephrolithiasis requiring multiple ureteral stent placements, for which she used tamsulosin 0.4 mg orally nightly; potassium citrate extended‐release 10 mEq orally twice daily had recently been stopped. She had Type 2 diabetes mellitus in remission following Roux‐en‐Y gastric bypass and was prescribed semaglutide (Ozempic) 1 mg subcutaneously once weekly; hypertension treated with lisinopril 10 mg orally daily; a prior transient ischemic attack with aspirin 81 mg orally daily; hyperlipidemia treated with rosuvastatin 5 mg orally daily; and obsessive‐compulsive disorder treated with clomipramine 50 mg orally nightly. Her history also included nonalcoholic fatty liver disease, obstructive sleep apnea without CPAP use, thalassemia trait, and gastroesophageal reflux disease, with omeprazole 20 mg orally daily listed but not recently taken. Other listed medications were ferrous sulfate 325 mg orally every other day and cholecalciferol 1000 units orally daily. Semaglutide was held during hospitalization, and lisinopril was held because of concern for acute renal dysfunction. During preoperative evaluation, SpO_2_ was 88% on room air. Despite escalation to 8 L/min supplemental oxygen via nasal cannula, SpO_2_ improved only to 92%, prompting cancellation of the procedure and hospital admission for further evaluation.

She had completed a 14‐day course of phenazopyridine 200 mg orally three times daily for dysuria after ureteral stent placement. She reported medication adherence and took her last dose on Day −1. Stent placement was estimated to have occurred on Day −15 by subtracting 14 days from the last dose; the procedure date was not independently verified. She had felt unwell for approximately 1 week and reported intermittent dizziness and bilateral upper extremity tingling, particularly with deep inspiration. She denied dyspnea, chest pain, cough, wheezing, fever, orthopnea, lower extremity edema, or decreased exercise tolerance. She had no history of chronic pulmonary disease and denied smoking or vaping. After ascorbic acid was started in the emergency department on Day 0 for suspected methemoglobinemia, oxygen was reduced to 4 L/min by nasal cannula. At subsequent inpatient assessment, she was comfortable and hemodynamically stable, with SpO_2_ of 93%–94%, respiratory rate of 14/min, heart rate of 64 beats/min, and blood pressure of 134/73 mmHg. Her lungs were clear, and neurological examination showed no focal deficits.

## 3 Methods

Initial testing showed normocytic anemia, with hemoglobin of 9.0 g/dL compared with 12.6 g/dL on Day −22. Haptoglobin was below 10 mg/dL, and lactate dehydrogenase (LDH) was at the upper limit of normal at 214 U/L. Total and direct bilirubin were 0.7 and 0.3 mg/dL, respectively. Creatinine was 1.57 mg/dL on Day 0 and 1.43 mg/dL on Day +1, compared with a baseline of 0.98 mg/dL recorded in an undated note. This increase supported acute renal dysfunction, although the undated baseline limited formal staging. There was no preexisting chronic kidney disease. The treating team attributed transaminitis to phenazopyridine; reported values included aspartate aminotransferase (AST) 31 U/L, alanine aminotransferase (ALT) 45 U/L, and alkaline phosphatase 129 U/L. The peripheral smear showed no bite cells, schistocytes, or spherocytes. Glucose‐6‐phosphate dehydrogenase (G6PD) testing, a reticulocyte count, and direct antiglobulin testing were not performed. Chest radiography and computed tomography angiography on Day 0 showed no acute cardiopulmonary abnormality or pulmonary embolism. Venous blood appeared chocolate brown, prompting consideration of dyshemoglobinemia.

The initial diagnosis was phenazopyridine‐associated methemoglobinemia with suspected oxidative hemolytic anemia. Phenazopyridine was discontinued; the last dose had been taken on Day −1. Ascorbic acid 500 mg orally once daily was started in the emergency department on Day 0, before oxygen flow was reduced from 8 to 4 L/min. The interval between the first dose and oxygen reduction was not charted. Serial venous blood gas tests from Day 0 through Day +3 repeatedly yielded indeterminate methemoglobin results; Figure 1 shows results from Days +2 and +3. Clomipramine was temporarily held while methylene blue was considered because of the risk of serotonin syndrome with concomitant use.

**FIGURE 1:**
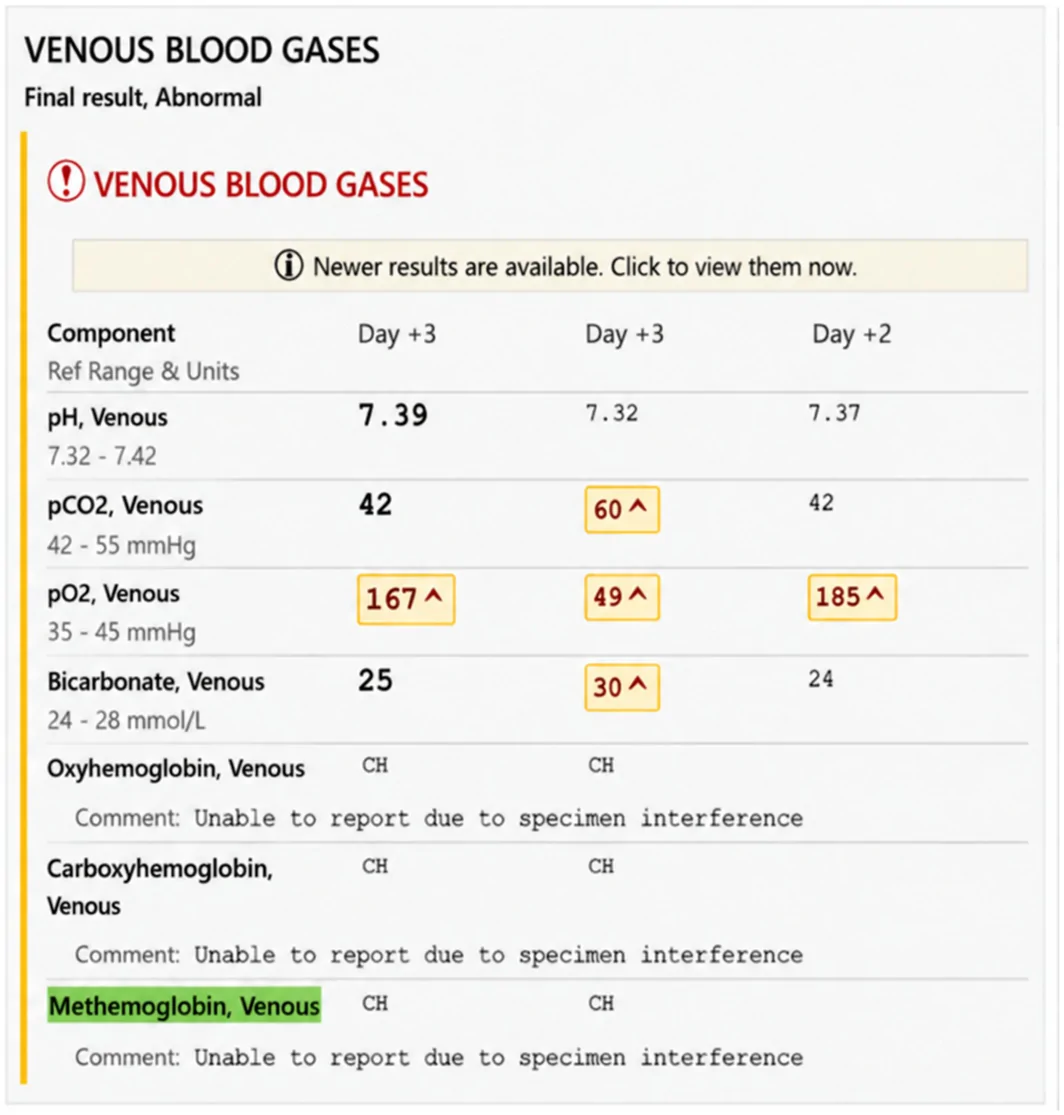
Serial venous blood gas results from Days +2 and +3. From left to right, the displayed columns correspond to Day +3, Day +3, and Day +2, with venous pO_2_ values of 167, 49, and 185 mmHg, respectively. The Day +3 columns show oxyhemoglobin, carboxyhemoglobin, and methemoglobin as unreportable because of specimen interference. The laboratory subsequently attributed the interference to sulfhemoglobinemia during its call on Day +5. These venous pO_2_ values are not arterial PaO_2_ measurements. Collection times are not shown in the image.

The first reliable methemoglobin result was 4% on Day +4. On Day +5, before discharge, the laboratory informed the treating team that sulfhemoglobinemia was causing the analyzer interference. The diagnosis was revised to phenazopyridine‐associated sulfhemoglobinemia based on this laboratory interpretation. A quantitative sulfhemoglobin concentration was not available.

## 4 Conclusion and Results

Hemoglobin was 9.0 g/dL on admission (Day 0), 8.7 g/dL later that day, and 9.5 g/dL on Day +1. Oxygen was gradually weaned from 4 L/min to room air, and dizziness and upper extremity paresthesias resolved. On Day +4, before transfusion, room‐air SpO_2_ was approximately 90%–91% and hemoglobin was 8.8 g/dL. The final predischarge creatinine was unavailable.

She received one unit of packed red blood cells on the night of Day +4 to augment oxygen‐carrying capacity in the setting of anemia and sulfhemoglobinemia. On Day +5, hemoglobin was 11.3 g/dL, room‐air SpO_2_ was 95%–96%, and she ambulated without difficulty. Methylene blue was not administered. Ascorbic acid was stopped at discharge because of her recurrent calcium oxalate nephrolithiasis and concern that supplementation could increase urinary oxalate [4, 5]. Clomipramine was restarted. She was discharged on Day +5 with plans to monitor hemoglobin, renal function, liver enzymes, and oxygen saturation. No postdischarge follow‐up data were available, and sulfhemoglobin clearance was not documented. Table 1 summarizes the hospital course.

**TABLE 1:** Hospital course timeline.

Day relative to presentation	Clinical event
Day −15 (estimated)	Estimated stent placement calculated as 14 days before the last dose on Day −1; not independently verified from a procedure record. Phenazopyridine 200 mg three times daily for a 14‐day course.
Day −1	Last documented phenazopyridine dose.
Day 0	Preoperative SpO_2_ 88% on room air; up to 8 L/min oxygen yielded SpO_2_ 92%. Procedure canceled and patient admitted. Chest imaging unrevealing; methemoglobinemia initially suspected. Creatinine 1.57 mg/dL. Ascorbic acid 500 mg orally daily started. The decrease to 4 L/min occurred after ascorbic acid was initiated in the emergency department; the exact titration interval was not charted.
Days 0 to +3	Hemoglobin 9.0 then 8.7 g/dL on Day 0 and 9.5 g/dL on Day +1. Creatinine 1.43 mg/dL on Day +1. Serial blood gas testing yielded indeterminate methemoglobin results. Clinically stable on 4 L/min with SpO_2_ 93%–94%; oxygen subsequently weaned.
Day +4	First reliable methemoglobin result: 4%. Pretransfusion hemoglobin 8.8 g/dL. One unit of packed red blood cells transfused that night.
Day +5	Posttransfusion hemoglobin 11.3 g/dL before discharge; room‐air SpO_2_ 95%–96%. Laboratory call identified sulfhemoglobinemia as the cause of analyzer interference. Discharged; ascorbic acid stopped and clomipramine restarted. No follow‐up data.

*Note:* Day 0 denotes presentation and hospital admission. Negative days precede presentation and positive days follow it.

Abbreviations: g/dL, grams per deciliter; L/min, liters per minute; mg, milligrams; mg/dL, milligrams per deciliter; SpO_2_, peripheral oxygen saturation measured by pulse oximetry.

## 5 Discussion

In this patient, sulfhemoglobinemia developed after a 14‐day course of phenazopyridine and was accompanied by suspected oxidative hemolysis, acute renal dysfunction, and transaminitis clinically attributed to the drug. Although the daily dose was within the usual adult range, the prolonged exposure raised concern for toxicity. Phenazopyridine labeling describes hemolytic anemia and renal and hepatic toxicity [1]. The patient had no preexisting chronic kidney disease, and impaired renal clearance before treatment was not established.

Laboratory consultation clarified the indeterminate co‐oximetry results. Sulfhemoglobin and methemoglobin have overlapping spectral characteristics, and some co‐oximeters cannot reliably distinguish them [3].

An indeterminate or discordant result should therefore prompt discussion of the analyzer's capabilities with the laboratory and consideration of specialized testing when available [3].

Conventional two‐wavelength pulse oximetry and routine blood gas co‐oximetry both have limitations in dyshemoglobinemia. Pulse oximetry estimates arterial oxygen saturation rather than tissue oxygen delivery, and dyshemoglobins can distort SpO_2_ [6]. Routine co‐oximetry may also fail to distinguish or quantify sulfhemoglobin [3].

Preoperatively, the patient received up to 8 L/min oxygen to achieve SpO_2_ of 92%. After ascorbic acid was started in the emergency department on Day 0, she remained comfortable and hemodynamically stable on 4 L/min, with SpO_2_ of 93%–94%. Her clinical stability supported continued care at the lower flow. The titration interval was not charted, so the reduction cannot be attributed specifically to ascorbic acid. Experimental methemoglobinemia can drive conventional pulse oximeter readings toward approximately 85% [7], and other dyshemoglobins can also affect these measurements [6]. This general behavior does not establish the cause of her changing SpO_2_ readings, particularly because the first reliable methemoglobin result was only 4% and no sulfhemoglobin concentration was available.

Chocolate‐brown venous blood provided an additional bedside clue to dyshemoglobinemia (Figure 2). This observation, together with persistently low SpO_2_ and unrevealing chest radiography and computed tomography angiography, prompted further evaluation. The discoloration was a clue rather than a definitive test; differentiation of sulfhemoglobin from methemoglobin requires appropriate laboratory evaluation [3, 8].

**FIGURE 2:**
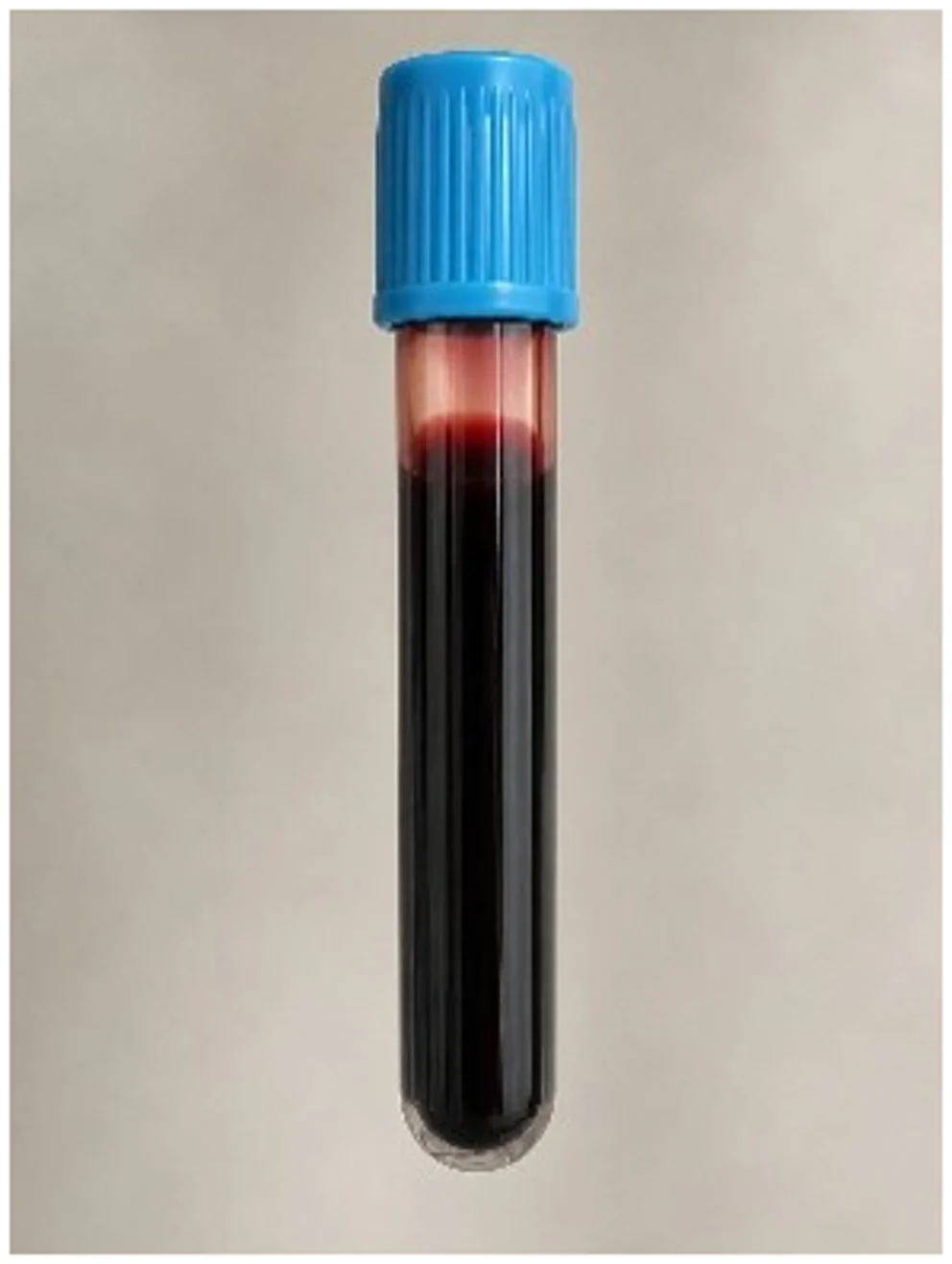
Dark chocolate‐brown blood specimen obtained during the patient's evaluation. The discoloration provided a bedside clue to dyshemoglobinemia but did not independently distinguish methemoglobinemia from sulfhemoglobinemia.

The fall in hemoglobin from 12.6 to 9.0 g/dL and haptoglobin below 10 mg/dL suggested hemolysis, although LDH was only at the upper limit of normal and bilirubin was normal. Oxidative hemolysis is a recognized adverse effect of phenazopyridine [1]. These findings alone do not establish the mechanism in this patient.

No bite cells, schistocytes, or spherocytes were seen on the peripheral smear. We did not obtain a supravital stain, reticulocyte count, direct antiglobulin test, or G6PD assay. G6PD deficiency predisposes to acute hemolysis triggered by exogenous agents [9]. Oxidative hemolysis therefore remained a clinical suspicion, and an underlying predisposition was not excluded.

Retrospective assessment with the Naranjo Adverse Drug Reaction Probability Scale yielded 7 points, indicating a probable adverse reaction: prior reports (+1), onset after exposure (+2), improvement after withdrawal (+1), alternative causes judged unlikely (+2), and objective laboratory evidence (+1); all other items scored 0 [10]. The assessment of alternative causes is a clinical judgment: rating this item as unknown would reduce the score to 5, which remains in the probable category [10]. The scale does not prove that phenazopyridine caused every accompanying abnormality. Rechallenge, drug levels, and dose–response data were unavailable. Oxygen therapy and transfusion also limit attribution of improvement to drug withdrawal alone.

Dizziness and bilateral upper extremity paresthesias began before admission and resolved during hospitalization. Anemia can worsen symptoms of methemoglobinemia by further reducing oxygen delivery [8]. This provides a possible physiological explanation for symptoms in a patient with both anemia and dyshemoglobinemia, but it does not establish peripheral nerve hypoxia as the cause of her paresthesias. The first reliable methemoglobin result, 4%, was obtained on Day +4; earlier levels were indeterminate. The mechanism of her symptoms remains uncertain, and other explanations were not excluded.

Treatment consisted of phenazopyridine withdrawal and supportive care. Methylene blue does not reverse sulfhemoglobinemia [2, 3]. It was not given in this case, as the patient improved with drug withdrawal and supportive care. Clomipramine use also raised concern for serotonin syndrome with intravenous methylene blue, an interaction described in its prescribing information [11]. Clomipramine was held while methylene blue was considered and restarted at discharge. Methylene blue is used for clinically significant methemoglobinemia; this activity does not extend to the sulfhemoglobin component [3, 8].

Ascorbic acid can reduce methemoglobin and is used in selected cases of methemoglobinemia [8]. It was started for the initial suspicion of methemoglobinemia in this patient. She received 500 mg orally daily from Day 0 until discharge on Day +5. Its metabolism to oxalate was a concern because of her recurrent calcium oxalate stones. Studies using 2 g/day supplementation demonstrate that vitamin C can increase urinary oxalate [5, 12]. The cited AUA guideline advises avoiding vitamin C supplements that increase urinary oxalate in calcium oxalate stone formers [4]. These studies do not quantify risk at 500 mg/day. The initial dose was not documented as having been selected for stone risk; her stone history was the reason for discontinuation at discharge.

Transfusion provides additional functional hemoglobin but does not reverse sulfhemoglobin already present in erythrocytes [3]. After one unit of packed red blood cells on Day +4, hemoglobin was 11.3 g/dL on Day +5 and room‐air SpO_2_ was 95%–96%.

Clinical recovery at discharge did not demonstrate sulfhemoglobin clearance. Without serial sulfhemoglobin measurements or postdischarge follow‐up, we could not assess clearance independent of transfusion. The undated baseline creatinine and unavailable final predischarge creatinine also limited assessment of renal recovery. Incomplete hemolysis testing prevented confirmation of the proposed oxidative mechanism.

Prior reports demonstrate the diagnostic difficulties associated with phenazopyridine‐induced sulfhemoglobinemia. Gopalachar et al. [2] described a 63‐year‐old woman whose diagnosis was confirmed after a lack of response to methylene blue. Morales et al. [13] described an 83‐year‐old woman with marked cyanosis and low SpO_2_ despite a high arterial oxygen tension, with sulfhemoglobin interference noted on co‐oximetry. These reports support consideration of sulfhemoglobinemia when oxygenation measurements and the clinical presentation appear discordant.

Phenazopyridine‐associated sulfhemoglobinemia should be considered when low SpO_2_ and indeterminate co‐oximetry remain unexplained. In this patient, laboratory consultation corrected the provisional diagnosis and guided supportive management. The concurrent anemia, renal dysfunction, and transaminitis warrant attention, while the cause of her neurological symptoms remains uncertain.

## Author Contributions

**Waldemar Irizarry:** writing – original draft, writing – review and editing, investigation, methodology. **Ashton Taylor:** writing – review and editing, methodology. **Amaya Arora:** writing – review and editing. **Astha Guliani:** conceptualization, investigation, writing – review and editing, methodology.

## Funding

The authors have nothing to report.

## Consent

Written informed consent was obtained from the patient for publication of this case report and accompanying images.

## Conflicts of Interest

The authors declare no conflicts of interest.

## Data Availability

Data sharing is not applicable to this article as no datasets were generated or analyzed during this study.
